# Supplementary material for: Study on the Mechanism of the Adrenaline-Evoked Procoagulant Response in Human Platelets
Source: Int J Mol Sci. 2024 Mar 5;25(5):2997. doi: 10.3390/ijms25052997 (PMC10932417; doi:10.3390/ijms25052997)
Supplement: Supplementary file 1 [file ijms-25-02997-s001.zip › ijms-2858563-supplementary.pdf]

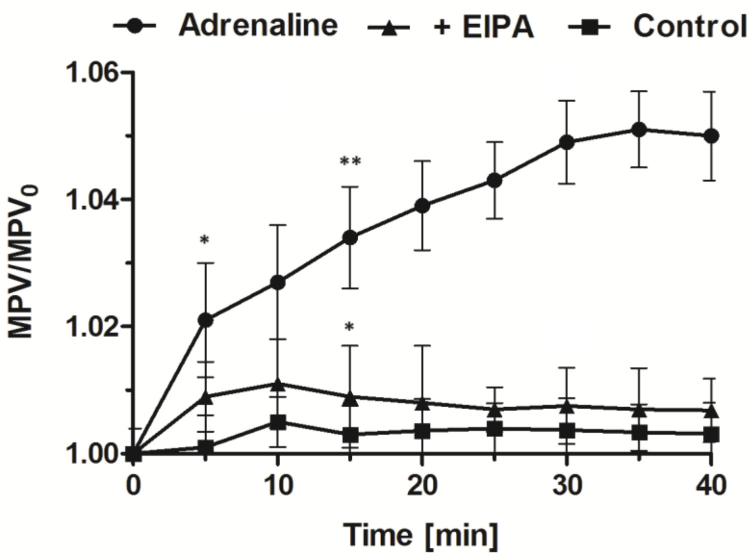

Figure S1. Dynamics of platelet volume exposed to adrenaline. Mean platelet volume (MPV) was measured by using electronic method. No decrease of platelet count was noted during incubation with adrenaline. The presented result (relative volume of platelet) was expressed as the ratio of MPV platelet stimulated by adrenaline to MPV control platelets. The values of MPV in control samples were between 7.2 and 8.3 fl. Adr – adrenaline. Presented values are means ± S.D. from 4 independent experiments. * $p < 0.05$, ** $p < 0.01$ vs control.
